# Site-directed mutagenesis reveals a unique requirement for tyrosine residues in IL-7Rα and TSLPR cytoplasmic domains in TSLP-dependent cell proliferation

**DOI:** 10.1186/1471-2172-11-5

**Published:** 2010-02-08

**Authors:** Jun Zhong, Akhilesh Pandey

**Affiliations:** 1 McKusick-Nathans Institute of Genetic Medicine and Departments of Biological Chemistry, Oncology and Pathology, Johns Hopkins University School of Medicine, 733 N Broadway, Baltimore, Maryland, 21205, USA

## Abstract

**Background:**

Thymic stromal lymphopoietin (TSLP) is an interleukin-7 (IL-7) like cytokine, which plays an important role in the regulation of immune responses to allergens. TSLP binds to a heterodimeric receptor complex composed of the IL-7 receptor α chain (IL-7Rα) and the TSLP receptor (TSLPR, also known as CRLF2). It has previously been suggested that the lone tyrosine residue in the mouse TSLPR cytoplasmic domain is required for cell proliferation using chimeric receptor systems. Also the role of tyrosine residues in the IL-7Rα cytoplasmic domain in TSLP signaling has not yet been investigated. We undertook a systematic analysis to test the role of tyrosine residues of both the IL-7Rα and the TSLPR in inducing cell proliferation in a growth factor dependent cell line, Ba/F3.

**Results:**

A multiple sequence alignment of the IL-7Rα and TSLPR cytoplasmic domains revealed conservation of most, but not all, cytoplasmic tyrosine residues across several species. Our site-directed mutagenesis experiments revealed that the single tyrosine residue in human TSLPR was not required for TSLP-dependent cell proliferation. It has previously been reported that Y449 of human IL-7Rα is required for IL-7 dependent proliferation. Interestingly, in contrast to IL-7 signaling, none of tyrosine residues in the human IL-7Rα cytoplasmic domain were required for TSLP-dependent cell proliferation in the presence of a wild type TSLPR. However, the mutation of all cytoplasmic four tyrosine residues of human IL-7Rα and human TSLPR to phenylalanine residues abolished the proliferative ability of the TSLP receptor complex in response to TSLP.

**Conclusion:**

These results suggest that TSLP requires at least one cytoplasmic tyrosine residue to transmit proliferative signals. Unlike other members of IL-2 cytokine family, tyrosine residues in IL-7Rα and TSLPR cytoplasmic domains play a redundant role in TSLP-mediated cell growth.

## Background

Thymic stromal lymphopoietin (TSLP) was first identified as a growth factor in the conditioned medium supernatant from the Z210R.1 thymic stromal cell line to support B cell proliferation *in vitro *[1,2]. TSLP is now known to play a key role in the initiation of asthma [3,4]. TSLP shares IL-7Rα with IL-7. TSLP signaling is mediated by a heterodimeric receptor complex, which consists of the interleukin-7 receptor α chain (IL-7Rα) and a unique TSLP-binding receptor (TSLPR), to transmit proliferative signals in cells [5-7]. IL-7 binds to a heterodimeric receptor complex, the IL-7Rα and the cytokine receptor common gamma chain (γ_c_), which is shared by IL-2, 4, 7, 9, 15 and 21. Both TSLP and IL-7 can activate the transcription factor STAT5. In the IL-7 receptor complex, IL-7Rα binds to Jak1 and γ_c _binds to Jak3 upon addition of IL-7. However, none of Jak kinases have been reported to be phosphorylated by the binding of TSLP to its receptor [2]. Previous studies showed that Y449 in the IL-7Rα cytoplasmic domain provides a docking site for PI-3K and STAT5 and is required for a proliferative signal by IL-7 signaling [8-10]. Further, the tyrosine residues of γ_c _are not required for IL-7-mediated cell proliferation [11]. Isaksen and colleagues observed that the lone tyrosine residue of the mouse TSLPR cytoplasmic domain is required for TSLP-mediated cell proliferation using chimeric receptors composed of the human GM-CSFR α chain extracellular domain fused to the mouse TSLPR transmembrane and cytoplasmic domains and the human GM-CSFR β chain extracellular domain fused to the mouse IL-7Rα transmembrane and cytoplasmic domains [12]. Brown et al. showed that anti-IL-7Rα antibodies inhibited TSLP-mediated proliferation of pre B-leukemia [13], indicating that both IL-7Rα and TSLPR contribute to TSLP-dependent cell proliferation.

We aligned the protein sequences of IL-7Rα and TSLPR cytoplasmic domains and observed that Y449 and Y456 of human IL-7Rα and Y368 of human TSLPR were conserved across the species examined while Y401 of human IL-7Rα was not conserved. Because the role of tyrosine residues in the context of the 'native' form TSLP receptor complex in TSLP-mediated cell proliferation has not been previously investigated, we took a systematic approach to evaluate the role of cytoplasmic tyrosine residues of TSLP receptor complex in mediating TSLP-induced cell proliferation. Our data show that the cytoplasmic tyrosine residues of either human IL-7Rα or human TSLPR can mediate TSLP-induced cell proliferation and that mutation of all the four cytoplasmic tyrosine residues of human IL-7Rα and human TSLPR to phenylalanine residues is required to abolish TSLP-dependent cell proliferation.

## Results and Discussion

### Most, but not all, cytoplasmic tyrosine residues of IL-7Rα and TSLPR are conserved across species

**Figure 1:**
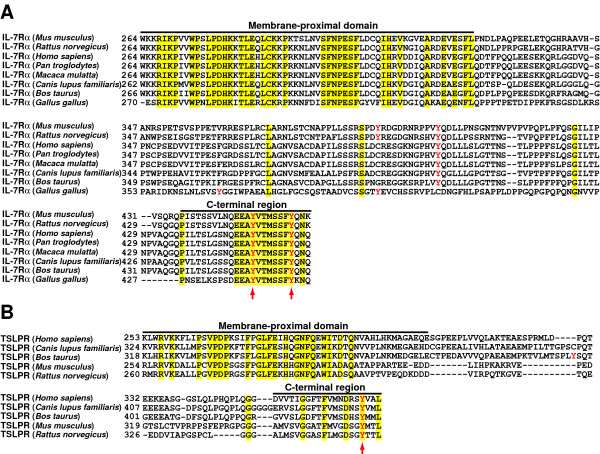
**Conservation of cytoplasmic tyrosine residues in IL-7Rα/TSLPR across species**. Sequence alignments of (A) the IL-7Rα cytoplasmic domains from *Mus musculus*, *Rattus norvegicus*, *Homo sapiens*, *Pan troglodytes*, *Macaca mulatta*, *Canis lupus familiaris*, *Bos taurus*, and *Gallus gallus *and (B) the TSLPR cytoplasmic domains from *Homo sapiens*, *Canis lupus familiaris*, *Bos taurus*, *Mus musculus*, and *Rattus norvegicus *were performed using ClustalX version 2.0.10 with the BLOSUM62 matrix. The conserved amino acid residues are highlighted in yellow while tyrosine residues are in red; conserved tyrosine residues are indicated by a red arrow.

Tyrosine residues in cytokine receptor cytoplasmic domains play important roles in mediating cytokine signaling and are mostly conserved across species. We aligned the amino acid sequences of IL-7Rα and TSLPR cytoplasmic domains from various species. Figure 1A shows an alignment of the IL-7Rα cytoplasmic domains. The membrane-proximal domains and C-terminal regions harboring two tyrosine residues are conserved among all IL-7Rα chains while the regions in between vary to some extent. The IL-7Rα cytoplasmic domains in *Mus musculus*, *Rattus norvegicus*, and *Gallus gallus *have four tyrosine residues while the IL-7Rα cytoplasmic domains in *Homo sapiens*, *Pan troglodytes*, *Macaca mulatta*, *Canis lupus familiaris *and *Bos taurus *only harbor three tyrosine residues. As indicated by red arrows in Figure 1A, the two tyrosine residues in the C-terminal region of the IL-7Rα chains are conserved while other tyrosine residues in the variable region are not conserved (red triangles). Figure 1B shows an alignment of the TSLPR cytoplasmic domains. As observed in the case of IL-7Rα, the membrane-proximal domains and C-terminal regions harboring one tyrosine residue are conserved in all TSLPRs while the intervening regions are variable. TSLPR cytoplasmic domains in *Homo sapiens*, *Mus musculus*, *Rattus norvegicus *and *Canis lupus familiaris *have only one tyrosine residue while the TSLPR cytoplasmic domain in *Bos taurus *harbors two tyrosine residues. Interestingly, the tyrosine residue in the C-terminal region of the TSLPR cytoplasmic domains across species is also conserved (a red arrow in Figure 1B). Because tyrosine residues play an important role in mediating the signaling by cytokine receptors and are conserved in the IL-7Rα and TSLPR cytoplasmic domains, we wanted to examine the role of these tyrosine residues in TSLP-dependent cell proliferation.

### The lone cytoplasmic tyrosine residue in TSLPR is not required for TSLP-dependent cell proliferation

**Figure 2:**
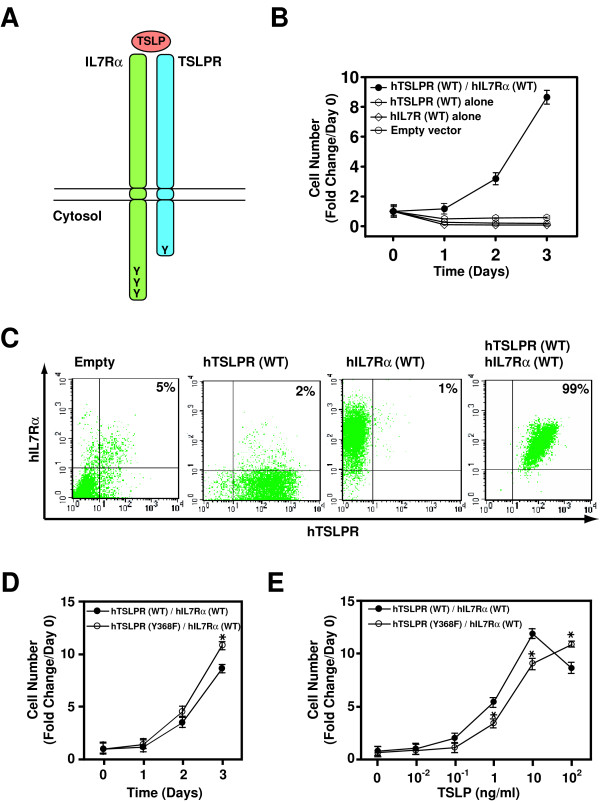
**The lone cytoplasmic tyrosine residue of human TSLPR is not required for TSLP-dependent cell proliferation**. (A) A schematic illustration of the human TSLP receptor complex composed of the human IL-7Rα and the human TSLPR. Y denotes the cytoplasmic tyrosine residues. (B) Exponentially growing Ba/F3 cells were starved and then treated with human TSLP (100 ng/ml). The data are represented as fold changes with the number of cells seeded on day 0 (for TSLP stimulation) representing the baseline. The mean of experiments done in triplicate for each time point and each cell line is shown. Error bars indicate S.E.M. (C) Surface expression of the wild type human TSLPR and IL-7Rα in the cell lines used in (B). Exponentially growing infected Ba/F3 cells were analyzed by flow cytometry for cell surface expression of human TSLPR and IL-7Rα. (D) TSLP-induced proliferation of Ba/F3 cells co-expressing the indicated were examined as described in (B). Statistical significance of the proliferative responses in the different cells at the same time point was evaluated using unpaired Student's t-test (* indicates a significant difference with p-value < 0.01). (E). Exponentially growing Ba/F3 cells were starved and then stimulated with the indicated concentration of human TSLP for 3 days. Cell numbers were counted after 3 days. The data are represented as fold changes with the number of cells seeded on day 0 (for TSLP stimulation) representing the baseline. Statistical significance of the proliferative responses in the different cells in response to the same dose of TSLP was evaluated using unpaired Student's t-test (* indicates a significant difference with p-value < 0.01). (The growth of cells was examined in 3 independent sets of experiments and found to be similar. The results from one representative experiment are shown).

Cytokines IL-2, 4, 7, 9, 15 and 21 share the common receptor subunit γ_c _that shows high homology to TSLPR. It has been reported earlier that these cytokines do not require the tyrosine residues of the γ_c _cytoplasmic domain to support cell growth [11]. In contrast, Isaksen and colleagues reported earlier that the single tyrosine residue of the mouse TSLPR cytoplasmic domain is critical for TSLP-dependent cell proliferation [12]. Because the study was based on a chimeric receptor system, we sought to study the role of tyrosine residues in TSLP signaling in the context of the native TSLP receptor complex. TSLP requires the heterodimeric TSLP receptor complex - IL-7Rα and TSLPR - to transmit signals (Figure 2A). Reche et al. have shown that coexpressed human TSLPR and IL-7Rα receptor subunits respond to human but not mouse TSLP [7]. We retested the requirement of the receptor complex for human TSLP-mediated signaling in an IL-3 dependent mouse cell line, Ba/F3, which also expresses endogenous murine TSLPR. A retroviral system was used to generate Ba/F3 cells that express wild type hTSLPR and/or wild type hIL-7Rα. As shown in Figure 2B, only Ba/F3 cells expressing both hIL-7Rα and hTSLPR, but not those expressing hIL-7Rα or hTSLPR alone could proliferate in response to human TSLP. Cell surface expression of human TSLPR and human IL-7Rα was confirmed by flow cytometry analysis using anti-human TSLPR and anti-human IL-7Rα antibodies (Figure 2C). These results again confirmed the requirement of hIL-7Rα and hTSLPR for human TSLP action allowing us to use this system for a systematic analysis of the requirement of tyrosine residues.

Human TSLPR contains only one cytoplasmic tyrosine residue (Y368) very close to the carboxyl terminus (Figure 1A). To determine whether this residue is required for TSLP-mediated cell proliferation, it was replaced by a phenylalanine residue (Y368F). A Ba/F3 cell line expressing both hTSLPR (Y368F) and hIL7Rα (WT) was established using retrovirus-based infection. As shown in Figure 2D and 2E, mutation of this tyrosine residue failed to abolish the proliferative response to TSLP. On day 3 of culture, 100 ng/ml TSLP induced ~25% more proliferation in Ba/F3 cells expressing hTSLPR (Y368F)/hIL-7Rα (WT) than Ba/F3 cells expressing hTSLPR (WT)/hIL-7Rα (WT) (p-value < 0.01) (Figure 2D). To examine this in greater detail, we carried out a dose response study (Figure 2E). We observed that in contrast to what was observed at 100 ng/ml, Ba/F3 cells expressing the hTSLPR (Y368F)/hIL-7Rα (WT) pair grew at a slower rate (p-value < 0.01) than Ba/F3 cells expressing the hTSLPR (WT)/hIL-7Rα (WT) in response to low doses of TSLP (1 ng/ml and 10 ng/ml). There was no statistical difference between the two at 0.01 ng/ml and 0.1 ng/ml. The cell surface expression of human TSLPR and human IL-7Rα was similar across the cell lines as confirmed by FACS using anti-human TSLPR and anti-human IL-7Rα antibodies (data not shown). Nevertheless, in contrast to a previous report using a chimeric receptor system [12], our data showed that the lone tyrosine residue in the TSLPR cytoplasmic domain is not required for TSLP-dependent cell proliferation. Our findings suggest that studies about TSLP-mediated signaling pathways should be carried out in the context of the native TSLP receptor complex. Intriguingly, these data suggest that Y368 in the hTSLPR cytoplasmic domain may play an inhibitory role in TSLP signaling in response to high doses of TSLP while playing a positive role at lower doses.

### The cytoplasmic tyrosine residues in IL-7Rα are required for IL-7 but not TSLP-dependent cell proliferation

**Figure 3:**
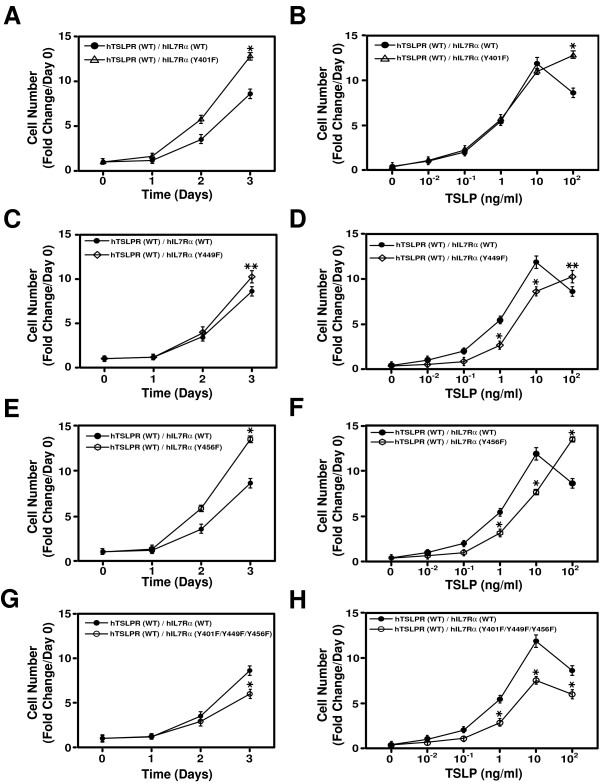
**The cytoplasmic tyrosine residues of the human IL-7Rα are not required for TSLP-dependent cell proliferation**. TSLP-induced proliferation of Ba/F3 cells co-expressing the indicated receptors was examined as described in Figure 2D. The proliferative response to different doses of TSLP was assessed in Ba/F3 cells expressing the indicated receptors as described in Figure 2E. Statistically significance was calculated using Student's t-test (* indicates a significant difference with p-value < 0.01; **p-value < 0.05). (The growth of cells was examined in 3 independent sets of experiments and found to be similar. The results from one representative experiment are shown).

Like TSLP, the receptor complexes for IL-4, 7, 9 and 21 are all heterodimers. Previous studies showed that Y497 of the human IL-4R is essential for IL-4 mediated proliferation [14] and that Y449 of the human IL-7Rα is required for IL-7 induced cell growth [8,9]. It has also been shown that IL-9 requires Y407 of the IL-9R to transmit proliferative signals and activate STATs [15] and that Y501 of the IL-21R is critical for maximal IL-21-mediated proliferation [16]. The human IL-7Rα contains three tyrosine residues in its cytoplasmic domain. Because the cytoplasmic tyrosine residue of human TSLPR is not required for cell proliferation and cytoplasmic tyrosine residues are critical for cytokine receptor signaling, we wanted to determine the requirement of the tyrosine residues in the IL-7α cytoplasmic domain for TSLP-dependent cell proliferation. We replaced the cytoplasmic tyrosine residues in IL-7Rα with phenylalanine residues to generate IL-7Rα mutants. First, we generated three individual mutants of human IL-7Rα- Y401F, Y449F and Y456F. Three Ba/F3 cell lines expressing the wild type TSLPR along with the three mutated IL-7Rα were established using retrovirus-based infection. Interestingly, none of the three mutations in the human IL-7Rα cytoplasmic domain abolished the proliferative response to TSLP administration (Figure 3). The individual mutations, Y401F (Figure 3A), Y449F (Figure 3C) and Y456F (Figure 3E), even led to an enhanced proliferative response at 100 ng/ml of TSLP. However, at lower doses of TSLP, Y449F (Figure 3D) and Y456F (Figure 3F), exhibited a diminished response (p-value < 0.01). Because the three tyrosine residues in hIL-7Rα may play redundant roles in supporting cell proliferation, we mutated all of them to phenylalanine residues (Y401F/Y449F/Y456F) and transduced this mutant receptor into Ba/F3 cells along with wild type human TSLPR. Strikingly, the mutation of all three cytoplasmic tyrosine residues of the human IL-7Rα to phenylalanine residues was still unable to abolish the proliferative response to TSLP (Figure 3G and 3H). The cell surface expression of mutant or wild type hIL-7Rα and hTSLPR was similar as evidenced by FACS (data not shown).

**Figure 4:**
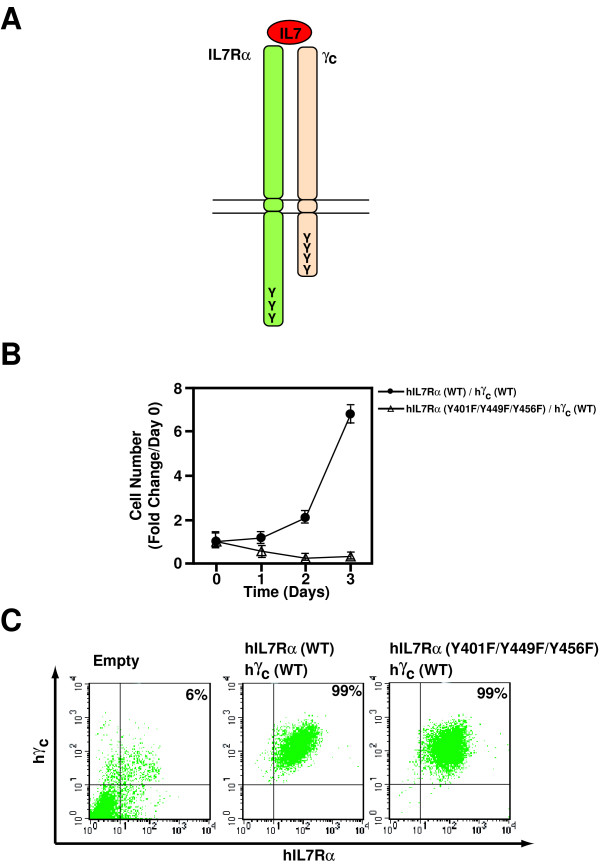
**The cytoplasmic tyrosine residues of the human IL-7Rα are required for IL-7-dependent cell proliferation**. (A) A schematic illustration of the human IL-7 receptor complex composed of the human IL-7Rα and the human γ_c_. Y denotes the cytoplasmic tyrosine residues. (B) IL-7-induced proliferation of various cell lines as indicated was examined as described in Fig. 2B. 100 ng/ml IL-7 was used. (C) Surface expression of the wild type/mutated human IL-7Rα and human γ_c _in the cell lines. Exponentially growing infected Ba/F3 cells were analyzed by flow cytometry for cell surface expression of human IL-7Rα and γ_c_. (The growth of cells was examined in 3 independent sets of experiments and found to be similar. The results from one representative experiment are shown).

Because the role of tyrosine residues in human IL-7Rα cytoplasmic domain in proliferation induced by IL-7, which requires IL-7Rα and γ_c _(Figure 4A), was established in a chimeric receptor system [8,9], we revisited this issue in Ba/F3 cells. Ba/F3 cells expressing the wild type or mutant human IL-7Rα (Y401F/Y449F/Y456F) and the wild type human γ_c _were established and evaluated for cell proliferation upon addition of human IL-7. In contrast to what we observed with TSLP, mutation of the three tyrosine residues in IL-7Rα completely abolished Ba/F3 cell proliferation in response to IL-7 (Figure 4B), which is consistent with previous reports [8,9]. Cell surface expression of human IL-7Rα and human γ_c _were similar by FACS using anti-human IL-7Rα and anti-human γ_c _antibodies (Figure 4C). Taken together, our data show that the cytoplasmic tyrosine residues in IL-7Rα are required for IL-7 but not TSLP-dependent cell proliferation.

### TSLP-dependent cell proliferation requires the presence of at least one tyrosine residue

**Figure 5:**
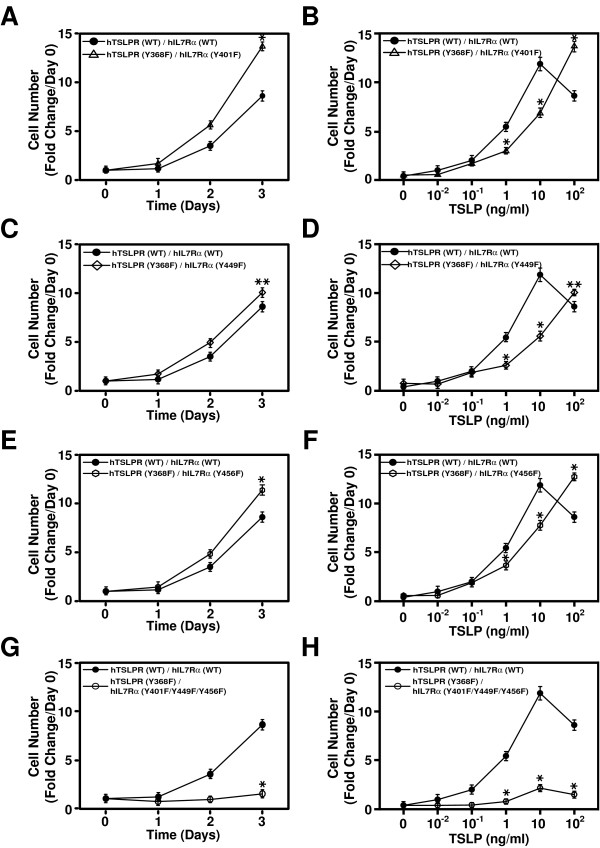
**TSLP-dependent cell proliferation requires the presence of at least one tyrosine residue**. TSLP-induced proliferation of Ba/F3 cells co-expressing the indicated receptors was examined as described in Figure 2D. The proliferative response to different doses of TSLP was assessed in Ba/F3 cells expressing the indicated receptors as described in Figure 2E. Statistically significance was calculated using Student's t-test (* indicates a significant difference with p-value < 0.01; **p-value < 0.05). (The growth of cells was examined in 3 independent sets of experiments and found to be similar. The results from one representative experiment are shown).

We next wanted to test whether any combination of mutation of tyrosine residues in IL-7Rα or TSLPR could affect TSLP-induced cell proliferation. For this, we generated three additional cell lines, hTSLPR (Y368F)/hIL-7Rα (Y401F), hTSLPR (Y368F)/hIL-7Rα (Y449F) and hTSLPR (Y368F)/hIL-7Rα (Y456F). As shown in Figure 5A, C and 5E, none of these combinations abolished cell proliferation in response to TSLP. As observed previously for individual tyrosine residues in IL-7Rα, combining them with TSLPR tyrosine mutants still led to a diminished response at low doses of TSLP (p-value < 0.01) (Figure 5B, D and 5F).

To test if any tyrosine residues were required for TSLP-induced proliferation, we mutated all tyrosine residues in the TSLP receptor complex (TSLPR (Y368F)/IL-7Rα (Y401F/Y449F/Y456F)). As shown in Figure 5G and 5H, this abolished the ability of the human TSLP receptor complex to drive cell proliferation. Taken together, our data suggest that TSLP signaling requires at least one tyrosine residue to support cell growth while all the four tyrosine residues in human TSLP receptor complex play various roles in TSLP-mediated cell proliferation.

### STAT5 phosphorylation requires at least one cytoplasmic tyrosine residue in the human TSLP receptor complex

**Figure 6:**
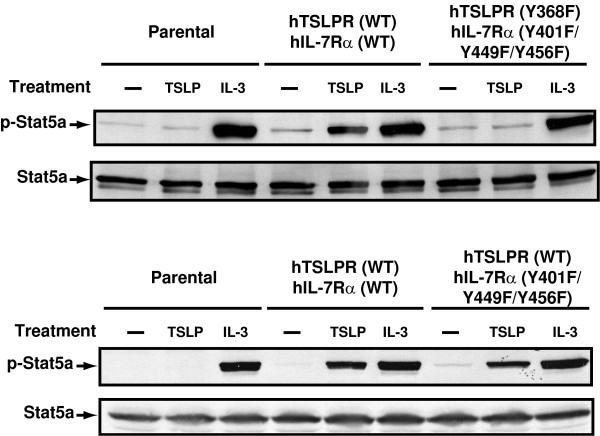
**TSLP-induced STAT5 phosphorylation requires the presence of at least one cytoplasmic tyrosine residue**. Exponentially growing Ba/F3 cells expressing the indicated receptors were washed three times with RPMI 1640, deprived of IL-3, and then left untreated or stimulated with recombinant human TSLP or mouse IL-3 as shown. After cell lysis, the cell lysates were subjected to immunoprecipitation using anti-STAT5a antibodies. After washing, the immunoprecipitates were resolved by SDS-PAGE. The phosphorylation status of STAT5a was assayed by Western blotting with anti-phosphotyrosine antibodies (4G10) and the total amount of Stat5a was measured by reprobing with anti-Stat5a antibodies as indicated.

Both human and mouse TSLP can induce STAT5 phosphorylation [2,7]. We examined whether TSLP-induced phosphorylation of STAT5 requires any of the cytoplasmic tyrosine residues of human TSLP receptor complex. As shown in Figure 6, while TSLP induced STAT5 phosphorylation in cells expressing wild type receptors and those expressing hTSLPR (WT)/hIL7Rα (Y401F/Y449F/Y456F), no phosphorylation was detectable in cells expressing hTSLPR (Y368F)/hIL7Rα (Y401F/Y449F/Y456F). Our data suggest that TSLP-induced STAT5 phosphorylation requires at least one cytoplasmic tyrosine residue in the human TSLP receptor complex. We also examined JAK2 phosphorylation in cells expressing wild type and various mutant receptors and failed to detect any tyrosine phosphorylation induced by TSLP (data not shown) in agreement with previous reports [2,17,18].

## Conclusions

Here we report that mutation of all four cytoplasmic tyrosine residues in the TSLP receptor complex abolishes the proliferative response to TSLP as well as STAT5 phosphorylation. Mutation of individual tyrosine residues in the cytoplasmic domain of human IL-7Rα or TSLPR was not sufficient to abolish this proliferative response although it did impair the response at low doses of TSLP (1 ng/ml and 10 ng/ml). Intriguingly, these mutations led to an increase in the proliferation rate in response to high doses of TSLP (100 ng/ml). Further experimentation will be required to elucidate the mechanistic basis of the signals that are mediated by the TSLP receptor complex. In this regard, it is interesting to note that the cytosolic tyrosine residues in IL-7Rα seem to be dispensable for TSLP, but not IL-7, signaling.

## Methods

### Reagents

PE-conjugated streptavidin, PE-conjugated anti-human CD4, PE-conjugated anti-human γ_c_, and Alexa Fluor^®^647 conjugated anti-human IL-7Rα antibodies were from BD Biosciences Pharmingen (San Jose, CA, USA). Anti-phosphotyrosine antibodies (HRP-conjugated) were from Millipore (Billerica, MA, USA) and anti-Stat5a antibodies were from Santa Cruz Biotechnology (Santa Cruz, CA, USA). Recombinant mouse IL-3, recombinant human IL-7, recombinant human TSLP and biotinylated anti-human TSLPR antibodies were from R&D Systems (Minneapolis, MN). RPMI 1640, fetal bovine serum (FBS), L-glutamine, and antibiotics were purchased from Invitrogen (Carlsbad, CA, USA). QuikChange XL-II mutagenesis kit was from Stratagene (La Jolla, CA, USA). Sequencing services were performed by the DNA and Peptide Synthesis and Sequencing facility at Johns Hopkins University School of Medicine using dye terminator chemistry. All other reagents used in this study were from Fisher Scientific (Pittsburgh, PA, USA).

### Cell culture

The interleukin-3 (IL-3)-dependent pre B cell line, Ba/F3, was grown in RPMI 1640 supplemented with FBS, L-glutamine, penicillin, streptomycin, and mouse recombinant IL-3 (10 ng/ml). Stably transfected Ba/F3 cell lines were grown in RPMI 1640 supplemented with heat-inactivated FBS, L-glutamine, penicillin, streptomycin and mouse recombinant IL-3 (10 ng/ml). Human embryonic kidney 293T (HEK293T) cells were cultured in Dulbecco modified essential medium, high glucose, supplemented with FBS, L-glutamine, penicillin, and streptomycin. Cell lines were maintained in the exponential growth phase unless indicated otherwise.

### Plasmids and expression vectors

The human TSLPR was obtained from Dr. James Ihle [19]. The human IL-7Rα was subcloned into a bicistronic retrovirus vector pMX-IRES-GFP [20] that expresses GFP while the human TSLPR and γ_c _were subcloned into a bicistronic retrovirus vector pMX-IRES-hCD4 [20] that expresses the human CD4 antigen. The human IL-7Rα, γ_c _and TSLPR mutants were generated by site-directed mutagenesis and confirmed by sequencing.

### Generation of stable cell lines using retroviruses

HEK293T cells were transfected with the retroviral vector constructs (12 μg) together with the helper virus pCL-ECO (12 μg) (Imegenex, San Diego, CA, USA) by using Lipofectamine 2000 (Invitrogen). Twenty-four hours after transfection, the supernatants were harvested and filtered through a 0.45-μm filter. Ba/F3 cells were infected with the pairs of the human IL-7Rα in pMX-IRES-GFP and the human TSLPR or γ_c _in pMX-IRES-hCD4. 48 hours after infection, Ba/F3 cells were stained by PE-conjugated anti-human CD4 antibodies and then sorted for GFP and the human CD4 expression.

### Receptors cell surface expression

After sorting, exponentially growing Ba/F3 cells expressing the pair of the wild type/mutated TSLP receptor complex were washed twice, stained with biotinylated anti-human TSLPR antibodies followed by PE-conjugated streptavidin and Alexa Fluor^®^647 conjugated anti-human IL-7Rα and analyzed by flow cytometry. Similarly, exponentially growing Ba/F3 cells expressing the pair of the wild type/mutated IL-7 receptor complex were washed twice, stained with PE-conjugated anti-human γ_c_, and Alexa Fluor^®^647 conjugated anti-human IL-7Rα antibodies and analyzed by flow cytometry.

### Immunoprecipitation and Western blotting

Exponentially growing Ba/F3 cells expressing different combinations of receptors were washed three times with RPMI 1640, deprived of IL-3 for 16 h, and then left untreated or stimulated with recombinant human TSLP or mouse IL-3 for 10 minutes at 37°C. Cells were lyzed in modified RIPA buffer (50 mM Tris-HCl, pH 7.4, 150 mM NaCl, 1 mM EDTA, 1% Nonidet P-40, 0.25% sodium deoxycholate, and 1 mM sodium orthovanadate in the presence of protease inhibitors) followed by centrifugation. The supernatant was subjected to immunoprecipitation using anti-Stat5a antibodies. After washing, the immunoprecipitates were resolved by SDS-PAGE and assayed by Western blotting with anti-phosphotyrosine antibodies (4G10) followed by reprobing with anti-Stat5a antibodies.

### Growth assays

The growth of cells expressing different combination of receptors was examined in 3 independent sets of experiments. In each experiment, exponentially growing Ba/F3 cells (in triplicate) expressing different combinations of receptors were washed three times with RPMI 1640, deprived of IL-3 for 16 h, and then stimulated with recombinant human TSLP or IL-7. Living cells were counted using a Beckman Coulter Z1 (Beckman Coulter, Fullerton, CA, USA). The results (except where indicated) are expressed as increase in cell number (stimulation [n-fold]) as compared to the number of cells plated on day 0. The mean and S.E.M were calculated for each independent experiment. The results from the three independent experiments were similar in each case and one representative experiment is shown in the figures.

### Protein sequence alignments

The protein sequence alignments were performed using ClustalX version 2.0.10 using the BLOSUM62 matrix.

### Statistical analysis

Data were expressed as mean ± SEM. Differences were examined by Student's t-test between two groups. p < 0.05 was considered significant.

## List of abbreviations

TSLP: thymical stromal lymphopoietin; IL-7Rα: interleukin-7 receptor α chain; TSLPR: TSLP receptor; γ_c_: interleukin-2 receptor γ chain/common γ chain.

## Competing interests

The authors declare that they have no competing interests.

## Authors' contributions

JZ and AP designed the experiments; JZ performed the experiments; JZ and AP drafted the manuscript. All authors read and approved the final manuscript.
